# Chronic *Escherichia coli* ST648 Infections in Patients with Cystic Fibrosis: The In Vitro Effects of an Antivirulence Agent

**DOI:** 10.3390/ijms26178650

**Published:** 2025-09-05

**Authors:** Olga L. Voronina, Marina S. Kunda, Natalia N. Ryzhova, Ekaterina I. Ermolova, Elizaveta R. Goncharova, Ekaterina A. Koroleva, Lidia N. Kapotina, Elena Yu. Morgunova, Elena L. Amelina, Nailya A. Zigangirova

**Affiliations:** 1 N.F. Gamaleya National Research Center for Epidemiology and Microbiology, Ministry of Health of Russia, Gamaleya Str., 18, 123098 Moscow, Russia; markunda99@gmail.com (M.S.K.); rynatalia@yandex.ru (N.N.R.); aksenova16@yandex.ru (E.I.E.); gon4arova.elizaveta@gmail.com (E.R.G.); korolevakate@yandex.ru (E.A.K.); lidiya.kapotina57@mail.ru (L.N.K.); lena.morgunova.1968@mail.ru (E.Y.M.); zigangirova@mail.ru (N.A.Z.); 2 Pulmonology Research Institute Under FMBA of Russia, Orekhovy Boulevard, 28, Building 10, 115682 Moscow, Russia; eamelina@mail.ru

**Keywords:** cystic fibrosis, *Escherichia coli* ST648, AMR, virulence factors, plasmids, transposons, Fluorthiazinone

## Abstract

Extraintestinal pathogenic *Escherichia coli* causes community-acquired and nosocomial pneumonia and poses a risk of infection, especially for patients with impaired lung function, such individuals with cystic fibrosis (CF). When chronic infection develops, eradication of the pathogen is difficult even with aggressive antibacterial therapy and targeted CF treatment. A new agent, Fluorthiazinone (CL-55), an inhibitor of bacterial virulence, was registered in Russia in 2024. The aim of our study was to characterize the genomes of *E. coli* ST648 isolated from long-term-infected CF patients, describe virulence factors, and investigate the effect of CL-55 on two CF isolates in vitro. Comparison of the genomes of hypermucoviscous isolates showed that, in the presence of a large number of core genes, the isolates have adaptive differences both in their chromosomes and the composition and genes of their plasmidomes. Both isolates formed mature biofilms on an abiotic surface and were able to survive and proliferate inside macrophages. CL-55 in in vitro experiments was effective in suppressing *E. coli* ST648 in both the aggregate and intracellular states, allowing us to propose the use of Fluorthiazinone as a combinative therapy to facilitate eradication of pathogenic microorganisms in the respiratory tract in patients with CF.

## 1. Introduction

Extraintestinal pathogenic *E. coli* (ExPEC) is one of the pathogens found in patients with respiratory infections and sepsis, and has been reported in cases of community-acquired (CAP), nosocomial (NP), and, especially, ventilator-associated pneumonia (VAP) [1,2]. The proportion of patients with pneumonia caused by *E. coli* varies from 7.7% in CAP in the USA [3] to 19% in VAP in France [4]. For VAP cases in Germany, *E. coli* was the dominant isolate [5]. In 55% of cases, the strain of *E. coli* responsible for pneumonia belongs to the phylogenetic group B2 [4] (for example, the pandemic ExPEC ST131 clonal lineage [6]). Another international high-risk clonal lineage, ST648, belonging to phylogenetic group F, is young, nascent, and characterized by low genetic diversity and associated with the carriage of antimicrobial resistance (AMR) genes with increased virulence potential [7]. *E. coli* ST648 was identified in a wide range of hosts (including healthy and diseased humans, companion animals, livestock, and wild birds) and the environment [7,8,9,10,11,12,13,14,15,16,17,18,19,20]. A total of 131 isolates of *E. coli* ST648 have been deposited in the *Escherichia* spp. PubMLST database, comprising clinical, animal, and environmental sources from 20 countries on five continents [8]. In total, 80% of clinical isolates were ExPEC and were retrieved from cases of bacteremia [8]. Based on the application of a negative frequency-dependent selection model to the bacteremia sample set, Schaufler et al. predicted a 10-fold increase in the frequency of ExPEC ST648 as a cause of bacteremia over the next few years [7]. ExPEC ST648 is also associated with respiratory, urinary, and wound infections. These strains frequently encode antibiotic resistance factors, including ESBLs (like CTX-M), carbapenemases (NDM), and colistin resistance (MCR-1), making ExPEC ST648 a significant threat to public health [7,9,10,11,12,13,14,15,16,17,18,19,20]. It should be noted that according to the assessment of the global burden of AMR from 1990 to 2021, *E. coli* was among the top three causes of deaths among adults and children, after *Staphylococcus aureus* and *Acinetobacter baumannii*, due to resistance to aminopenicillins, beta-lactams and beta-lactamase inhibitors, fluoroquinolones, and third-generation cephalosporins [21].

The spread of pathogenic *E. coli* lineages increases the likelihood of infection for high-risk patients, such as patients with cystic fibrosis (CF), through exposure in the environment, the community, and hospitals. CF is a chronic genetic disorder caused by a mutation in the gene of the CF transmembrane conductance regulator (*cftr*), leading to systemic pathology. Chronic lung infections are important manifestations of CF, causing progressive airways disease and respiratory failure, the primary cause of mortality in CF patients [22]. *E. coli* is not a classical CF pathogen, which is likely why European and American CF registries do not register data on chronic *E. coli* infection. However, some information is available in the scientific literature. A seven-year study of 176 CF patients in two German CF centers revealed that 11% had chronic *E. coli* infection [23]. *E. coli* belonging to 11 ST (ST12, ST39, ST73, ST95, ST126, ST127, ST141, ST357, ST420, ST2015, and ST2628) caused long-term infection [23]. In Canada, over 38 years of observation of 366 CF patients, the proportion of patients with chronic *E. coli* infection (ST131, ST73, and ST1193) was 4.9%, and 2.4% of patients experienced exacerbation due to *E. coli* infection [24,25]. In the Moscow region, according to the registry of Russian patients with CF, the proportion of patients with chronic lung *E. coli* infection was 3.6% in 2023 [26]. In our study of the lung microbiome of 50 adult CF patients from the Moscow region, three patients had chronic *E. coli* infection: two patients were infected with *E. coli* ST648, and one patient was infected with *E. coli* ST1193. Thus, ExPEC ST648 has not previously been reported in the CF patient cohorts in published data.

Long-term persistence and difficulties in the eradication of *E. coli* are associated with the genetic characteristics of ExPEC strains, particularly those responsible for virulence potential. One of the especially important virulence determinants is the capsular polysaccharide (CPS), or K antigen. K antigens have been classified into four groups [27]. ExPEC associated with invasive disease usually express group 2 CPSs that include K1, K4, and K5 polysaccharides, among others [28]. The expression of this group of CPSs is temperature-regulated and switches inside the host at 37 °C [27]. ExPEC strains producing group 2 CPSs may also co-express colanic acid. Excessive production of these surface polysaccharides can result in a hypermucoviscous phenotype [29]. It has been shown that the hypermucoviscosity of *K. pneumoniae* isolates has also been associated with the development of invasive syndromes [30]. Some exceptional hypermucoviscous ExPEC strains have been described [29,31]. The hypermucoviscous phenotype decreases the host’s immunological defenses and enhances the bacterial survival rates [32].

*E. coli* infection is initiated via bacterial adhesion to the lung epithelial surface. Direct interactions between human interleukin-8 (IL-8) and the chemoreceptor Tsr expressed on the surface of *E. coli* play an important role in the transmigration of the bacterium across the human lung mucosal barrier via both paracytosis and transcytosis [33]. Several in vitro (cell culture) and in vivo (in mice) studies have shown that *E. coli* is able to invade alveolar epithelial cells type II [34] and persist in macrophages [35,36] and neutrophils [37]. As a result, the presence of AMR and virulence factors, the ability to form biofilms, and persistence within different types of eukaryotic cells allow *E. coli* to avoid the effects of maintenance and episodic antibiotic and CFTR-modulator therapy.

A new antivirulence drug, Fluorthiazinone (FT), has been developed based on a low-molecular lipophilic inhibitor of T3SS (C19H17F2N3O4S), CL-55 [38]. In terms of its mechanism of action, FT differs from antibiotics, since it does not kill bacteria, but suppresses the functioning of T3SS and the flagellum, which are structurally similar due to a common evolutionary origin. As has been experimentally shown on a number of representatives of Gram-negative bacteria, FT suppresses cytotoxicity, motility, invasion, intracellular survival in epithelial cells, and the formation of biofilms [39,40,41]. Suppression of virulence in the body leads to blocking of infection, which has been shown in model infections caused by various pathogenic bacteria of the kingdom *Pseudomonadati* [42]. In 2024, FT was registered in Russia after the completion of phase 3 clinical trials on patients with complicated urinary tract infections [42].

Expanding the list of diseases for which FT can be used involves studying the characteristics of microorganisms relevant to patients and their sensitivity to a new antibacterial drug. Bacteria that chronically infect patients with cystic fibrosis differ from bacteria that cause hospital-acquired and community-acquired infections [43].

So, the aim of our study was to compare the genomes of isolates obtained from two patients with *E. coli* ST648, describe AMR and virulence factors, and investigate the effect of FT on *E. coli* ST648 isolated from long-term infected CF patients in vitro to evaluate the impact of FT on characterized isolates.

## 2. Results

### 2.1. Microbiome of the Sputum Samples

**Figure 1:**
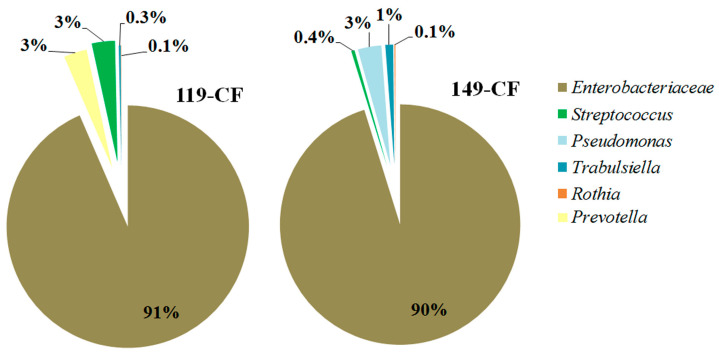
Microbiome composition of sputum samples from which *E. coli* GIMC1402:EC_33P15 (patient 119-CF) and GIMC1403:EC_33P43 (patient 149-CF) were isolated.

The sputum samples from long-term *E. coli*-infected patients, 119-CF and 149-CF, were analyzed for the presence of *E. coli* via amplification and sequencing of *16S rDNA* and *adk* gene fragments. The main pathogen was *E. coli*, and the proportion of *E. coli*, according to the analysis of the microbiome composition, was 91 and 90% in the samples of patients 119-CF and 149-CF, respectively (Figure 1). *Streptococcus* and *Prevotella* were present in small quantities in the 119-CF microbiome, and *Pseudomonas* and *Trabulsiella* were present in the 149-CF microbiome. These samples were used for *E. coli* isolation. The isolates GIMC1402:EC_33P15 (patient 119-CF) and GIMC1403:EC_33P43 (patient 149-CF) were primarily characterized using whole-genome sequencing.

### 2.2. Features of the Genomes of E. coli Isolates

Genomic analysis of isolates GIMC1402:EC_33P15 and GIMC1403:EC_33P43 showed that both isolates belonged to the phylogenetic group F, the international high-risk clone ST648, and had antigen profiles O153:H6:K5 (GIMC1402:EC_33P15) and O153:H6:K4 (GIMC1403:EC_33P43).

The main features of genome annotation are presented in Table 1. The GIMC1402:EC_33P15 chromosome is larger than the GIMC1403:EC_33P43 one and contains more CDSs (Coding DNA Sequences) and pseudogenes. Both genomes contain extrachromosomal DNA elements, represented by plasmids and phage-plasmids. The biggest are plasmid 1 and phage-plasmids, with a size of more than 100 kb, which are present in one copy per cell. The small plasmids with sizes of 2.1–7.2 kb have copy numbers of 10–20 per cell. All small plasmids encode Rep initiator proteins; some of them encode proteins required for conjugal mobilization and virulence factors, for example, VirB5, a minor pilin of the type IV secretion complex.

#### 2.2.1. Phage-Plasmids

**Figure 2:**
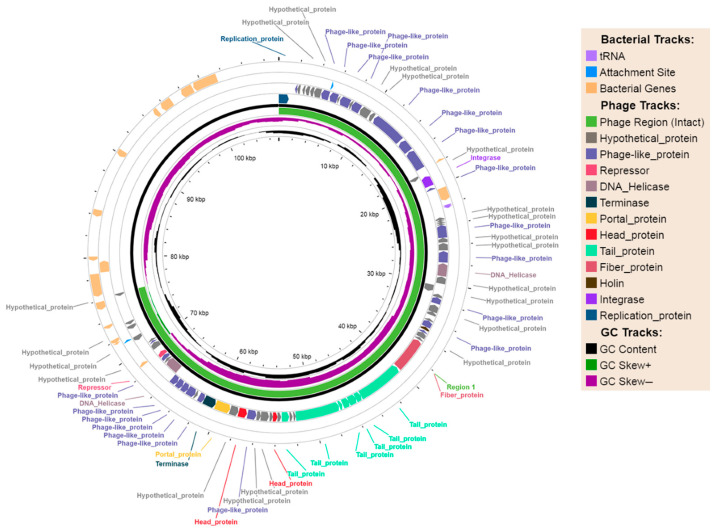
Phage-plasmid episome as part of the genomes of *E. coli* isolates GIMC1402:EC_33P15 and GIMC1403:EC_33P43.

Phage-plasmids (P-Ps) have a dual functionality: they can replicate independently as plasmids and carry prophage sequences. Under specific conditions, a release of intact phages can occur, which may lead to bacterial cell infection and death. P-Ps of the isolates GIMC1402:EC_33P15 and GIMC1403:EC_33P43 belong to the plasmid incompatibility group IncFIB. Based on genome-wide sequence similarity, these P-Ps are part of the SSU5 super-community of phage-plasmids associated with species in the *Enterobacteriaceae* family and belong to the pSLy3 group according to Pfeifer et al.’s classification [44]. Phage-plasmids contain intact prophage sequences of 76.2 kb in length (Figure 2) and the *repB* gene, which encodes plasmid replication initiator proteins; however, they do not carry any genes encoding AMR. It is interesting that both P-Ps acquired the *apbC* gene for the iron–sulfur cluster carrier protein ApbC. ApbC can bind and rapidly transfer iron–sulfur ([Fe-S]) clusters to an apoprotein and also demonstrates ATPase activity. ApbC is a member of the ParA subfamily of proteins, which have a wide array of functions, including electron transfer, initiation of cell division, and DNA segregation [45].

#### 2.2.2. The Large Plasmids pEC_33P15-1 and pEC_33P43-1

**Figure 3:**
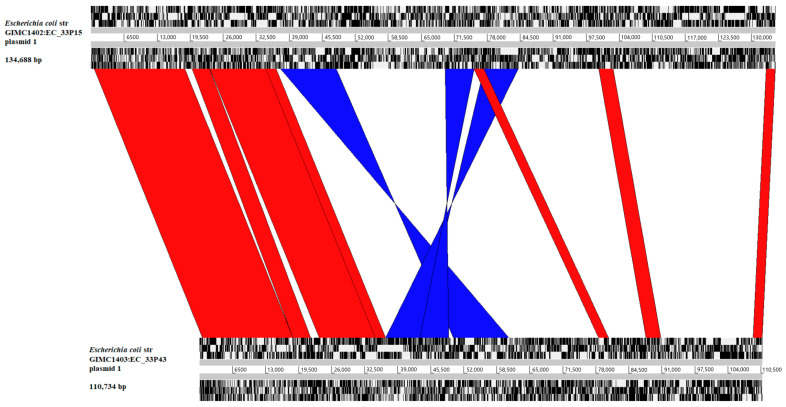
Complete DNA sequence plasmid comparisons. Upper scale: plasmid pEC_33P15-1 of isolate GIMC1402:EC_33P15; lower scale: plasmid pEC_33P43-1 of isolate GIMC1403:EC_33P43. Bands of color indicate homology between sequences. Red lines show sequence in the same confirmation; blue lines indicate sequence inversion.

The large plasmids of the isolates are characterized via IncFIB/IncFII/IncFIA and belong to the F family of conjugative plasmids. Complete nucleotide sequence comparison of pEC_33P15-1 and pEC_33P43-1 revealed regions of homology, three inverted regions, and regions of difference (Figure 3). The main regions of difference contained transposons and class 1 integrons that were present only in plasmid pEC_33P15-1. The regions included the following genes: *tet(B)* and *tetR(B)* (resistance to tetracyclines), *catA1* (chloramphenicols), *mph(A)* and *mrx(A)* (macrolides), *sul1* (sulfonamides), *qacE* (quaternary ammonium compounds), *aadA5* (aminoglycosides), and *dft17* (trimethoprim) (Table 2). The genes acquired by the plasmid enhance the potential for the isolate GIMC1402:EC_33P15 to develop resistance to different classes of antimicrobial drugs.

Analysis of virulence factors (Table 2) revealed that both plasmids carry genes encoding proteins that increase survival under conditions of divalent metal ion deficiency. These are ABC (ATP-binding cassette) and ILT (iron/lead transporter) transport systems that ensure efficient iron capture under conditions of limited iron availability, which contributes to the virulence and competitiveness of GIMC1402:EC_33P15 and GIMC1403:EC_33P43 isolates. In addition, ABC transporters are involved in the secretion of virulence factors and promote bacterial survival via efflux of toxic xenobiotics, which in turn contributes to antimicrobial resistance [47]. Aerobactin (*iucABCD*, *iutA*) clusters responsible for the synthesis of the siderophore that enables the capture and transport of iron from the environment, as well as the *sitABCD* transporter, were identified only in pEC_33P15-1. The *sitABCD* operon encoded an ABC transporter that transports divalent Fe^2+^ and Mn^2+^ cations, which not only promotes metal uptake but also enhances the resistance of the bacterium to oxidative stress. In addition, the *sitABCD* operon and the *qacE* gene identified in pEC_33P15-1 provide resistance to disinfectants [48,49]. Genes for aerobactin were absent in pEC_33P43-1. However, this plasmid carried the *yncE* gene and the gene encoding the TonB-dependent metal ion transport system, which were absent in pEC_33P15-1.

Another important virulence factor is the F-type transfer system, represented by several *tra* and *trb* genes and the *finO* gene, which are gathered in one 19.465 kb region in plasmid pEC_33P43-1. In plasmid pEC_33P15-1, this gene region is divided into two parts: 10,563 and 4968 kb, due to recombination and inversion. The F-type transfer system encodes the proteins involved in the elaboration of the conjugative pilus and the T4SS (type IV secretion system) required for the formation of the mating pair, as well as the relaxosome components needed for the processing of the plasmid prior to transfer [50]. Notably, the pilin TraA is of interest, as it is the main adhesion factor that induces biofilm formation, complementing flagella, type 1 fimbriae, Ag43, and curli, which are essential to *E. coli* biofilm [50]. So, the conjugative pilus of a derepressed F plasmid can promote the formation of biofilm in *E. coli* cells [51]. Moreover, the F plasmid, which does not express F pili, can induce the production of curli, which affects the maturation of the three-dimensional structure of the biofilm [52]. Another mechanism of enhancing biofilm formation under the influence of plasmids is mediated by their effect on reducing motility and increasing the level of quorum-sensing inducer AI-2 (auto inducer 2) [53]. Thus, the entire set of episomes contributes to biofilm formation via GIMC1402:EC_33P15 and GIMC1403:EC_33P43 isolates.

The next group of factors is the toxin–antitoxin systems. Two type I toxin–antitoxin systems (Mok/Hok, Hok/Gef) and two type II systems (CcdA/CcdB and PemL/PemK) are common for both plasmids (Table 2). The type II toxin–antitoxin system, VapB/VapC, is unique to pEC_33P15-1, and the Phd_YefM/Fic_DOC system is unique to pEC_33P43-1. The main function of toxin–antitoxin systems is to ensure the stability of plasmid inheritance and the formation of persister cells [54]. The presence of toxin–antitoxin determines the selective advantage of a clone in the bacterial population and the formation of a stable cell population. Thus, thanks to the plasmid, the isolate GIMC1402:EC_33P15 is characterized by more pronounced antibiotic resistance and an expanded set of virulence factors, which may provide it with advantages in antibiotic therapy and intermicrobial competition, whereas GIMC1403:EC_33P43 shows less potential for drug resistance.

#### 2.2.3. Comparative Analysis of Chromosomes

**Figure 4:**
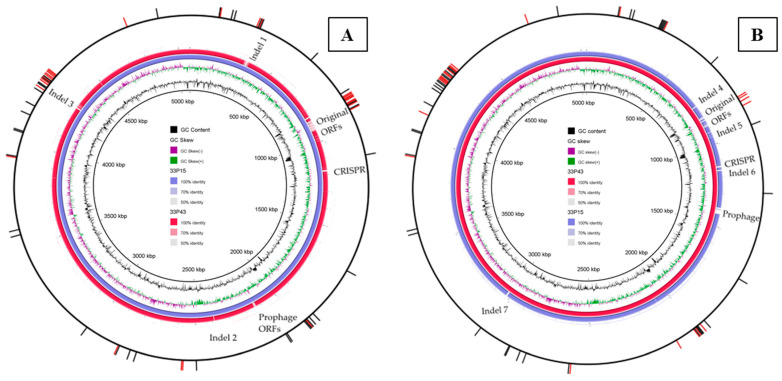
Comparative analysis of chromosomes of *E. coli* isolates GIMC1402:EC_33P15 and GIMC1403:EC_33P43. 1–7: number of regions of differences. (**A**) The query is GIMC1402:EC_33P15; (**B**) the query is GIMC1403:EC_33P43.

Comparative analyses of chromosomes revealed some regions of difference (Figure 4). First, they were associated with mobile genetic elements (MGEs): prophages (Figure 4) and transposons (Table 3). Two regions of transposons containing AMR determinants are presented in Table 3. Region 2, which includes the CTX-M-15 beta-lactamase ORF (open reading frame), is identical in the isolates. Region 1 contains ORFs present in both genomes (for aminoglycoside acetyltransferase AAC(6′)-Ib-cr and beta-lactamase OXA-1) and ORFs distinctive for GIMC1402:EC_33P15 (for aminoglycoside acetyltransferase AAC(3)-IIa, beta-lactamase TEM-1, and gene of the tunicamycin resistance protein TmrB). Region 1 is partly included in **Indel 1** (Figure 4, Table 4). In addition, Indel 1 contains the operon for the ABC transporter complex UgpBAEC involved in the import of sn-glycerol-3-phosphate (G3P), which is missing in GIMC1403:EC_33P43. G3P is an important intermediate in lipid biosynthesis and is a carbon source [55].

**Indel 2** contains four ORFs that are specific to GIMC1402:EC_33P15, including the ORF of the small-membrane protein Blr, which is capable of interacting with several divisomal proteins and stabilizing their assembly into a functional machinery [56]. This characteristic is also shared by YmgF, which is present in both isolates. However, an additional function of Blr is to increase the *E. coli* cell’s resistance to a wide spectrum of beta-lactam antibiotics or other drugs that inhibit peptidoglycan synthesis [57].

**Indel 3** was formed as a result of the duplication and recombination of individual regions in the chromosome of GIMC1402:EC_33P15. For example, ORFs for the type IV toxin–antitoxin system are repeated three times in the GIMC1402:EC_33P15 genome, while in the GIMC1403:EC_33P43 genome, they are present in one copy.

The marker operon for **Indel 4** is the operon for the tripartite ATP-independent periplasmic (TRAP) transporter, which uses energetically favorable cation gradients to drive the import of specific carboxylate- and sulfonate-containing nutrients against their concentration gradient [58]. Thus, in the GIMC1403:EC_33P43 genome, there are three TRAP operons, and in the GIMC1402:EC_33P15 genome, there are only two operons.

**Indel 5** includes ORFs of the energy-coupling factor (ECF)–ABC transporter for cobalt transport. This operon is absent in the GIMC1402:EC_33P15 genome.

**Indel 6** gathers genes for some metabolic pathways and an additional GntP family transporter (gluconate:H+ symporter) and is missing in the GIMC1402:EC_33P15 genome.

**Indel 7** includes 11 ORFs lacking in GIMC1402:EC_33P15. The most important of these for describing resistance and virulence factors are the *mdtH* gene encoding the multidrug efflux MFS transporter, and the biofilm formation regulator BssS. BssS, being a component of the GlaR regulon, is involved in the regulation of indole and uptake and export of AI-2 through a cAMP-dependent pathway; thus, BssS regulates biofilm through signal secretion [59].

Another indel is related to differences in the sequences of the **CRISPR-Cas regions** (Table 5). Both regions of the strain GIMC1402:EC_33P15 have the same repeat sequences but different numbers of spacers, so the CRISPR regions have different lengths and belong to different IDs according to the CRISPR database. CRISPR regions of the strain GIMC1403:EC_33P43 differ in both repeat sequences and the number of spacers. One of the regions has the same ID as the CRISPR region of the strain GIMC1402:EC_33P15; the other had no analogs in the database. In general, the length of the CRISPR regions in the genome of this strain was less than that of GIMC1402:EC_33P15. The sequences of the eight Cas protein genes were identical except for one encoding Cas 3.

**Figure 5:**
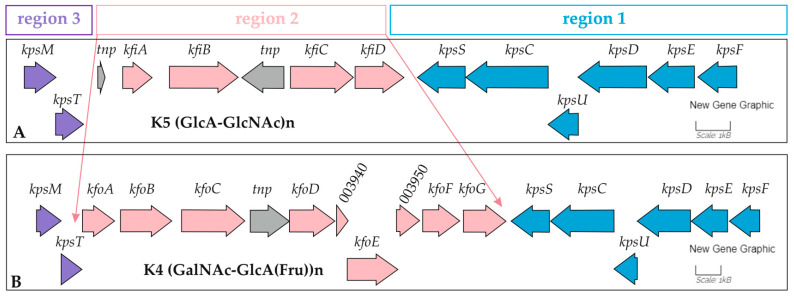
Organization of the genes required for the synthesis of K5 (**A**) and K4 (**B**) capsules. Regions 3 (violet) and 1 (blue) flanked region 2 (pink). ORFs are shown by arrows and are colored by region. ORFs of the transposases are colored gray.

The biggest one was **the region of the original ORFs** (Figure 4, Table 4). The formation of such a region was determined by differences in the K loci (Figure 5). The K loci of both strains belong to group 2, including conserved regions 1 and 3, and serotype-specific region 2. According to the structure of region 2, K5 strain GIMC1402:EC_33P15 and K4 strain GIMC1403:EC_33P43 synthesize a (GlcA-GlcNAc)n and a (GalNAc-GlcA(Fru))n polymers, respectively.

The second cluster of genes in the region of the original ORFs encodes proteins for metabolosome (bacterial microcompartment) organization and enzymes for propanediol utilization. GIMC1402:EC_33P15, containing this operon, benefits from the ability to utilize alternate carbon sources under conditions of inflammation.

Thus, a comparison of the chromosomes of two *E. coli* ST648 isolates showed that, despite the large number of core genes, the isolates differ in antigenic, metabolic, virulence, and resistance characteristics, reflecting the processes of adaptation of each isolate to the peculiarities of the respiratory tract of a particular patient.

The evidence of numerous introductions into genomes of GIMC1402:EC_33P15 and GIMC1403:EC_33P43 isolates is the presence of a large number of both functioning genes (40 and 32) and pseudogenes (24 and 30) of transposases. Most of them (51 and 49) were identified as transposases of 15 IS families. IS3, IS1, and IS66 were predominant. These introductions resulted in the acquisition of the CTX-M-15 gene, which was important for the evolution of *E. coli* ST648.

#### 2.2.4. The Place of CF Isolates in the Population Structure of *E. coli* ST648

The carriage of extended-spectrum beta-lactamase (ESBL) genes, especially CTX-M, is the hallmark of *E. coli* ST648. The strains of CF patients produced CTX-M-15 belonging to the CTX-M-1 group. CTX-M-15 was first observed in 1999 in an isolate from India, and now it is widespread around the world [60]. To roughly determine the time of infection of CF patients, we used the results of the analysis of the population structure of *E. coli* ST648, performed by Schaufler et al. for 87 strains isolated between 2006 and 2014 in different countries around the world [7]. The authors estimated that the earliest clades (3 and 4) separated from the latest clades (1 and 2) in 2004. The strains of clades 3 and 4 carried CTX-M of different alleles. The strains with CTX-M-15 belonged to clades 2 and 1. The split between clades 1 and 2 occurred in 2006.

We performed a pairwise alignment of the genomes of CF isolates and strains, representatives of clades 1–4, to calculate the ANI and AP values (Table 6). According to the data in Table 6, the ANI values were in the range of 98.84–99.72, which characterized an extremely closely related group of isolates within the species. According to the ANI and AP values, CF strains belonged to the latest clade 1 formed in 2006; therefore, with a certain degree of probability, the infection of patients occurred no earlier than 2006. The first molecular genetic confirmation of the genotype of *E. coli* infecting CF patients was obtained in 2013 for patient 119-CF and in 2015 for patient 149-CF.

#### 2.2.5. Adaptation to the Long-Term Chronic Infection

**Figure 6:**
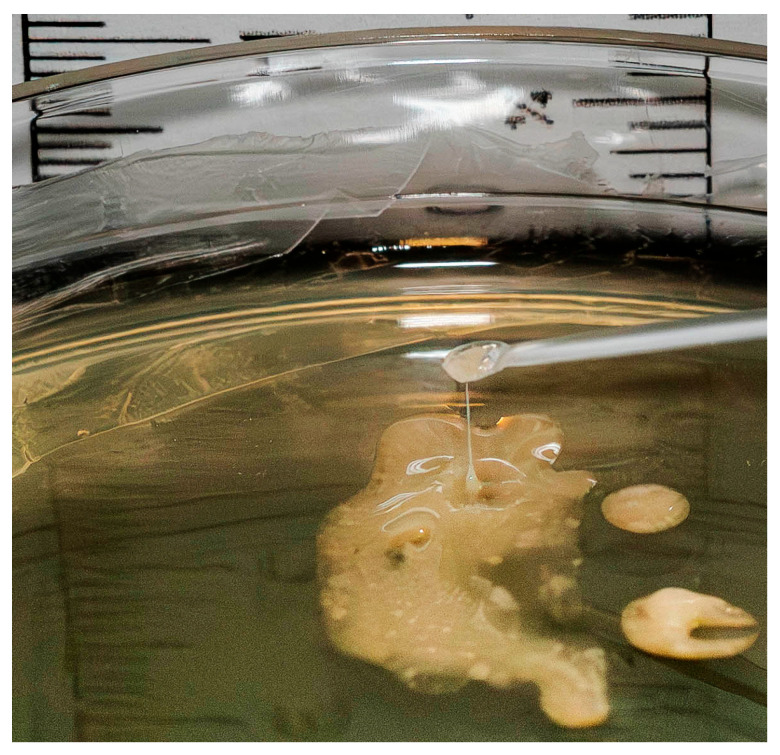
The hypermucoviscous colonies of the GIMC1402:EC_33P15 strain cultivated on LB agar.

**Figure 7:**
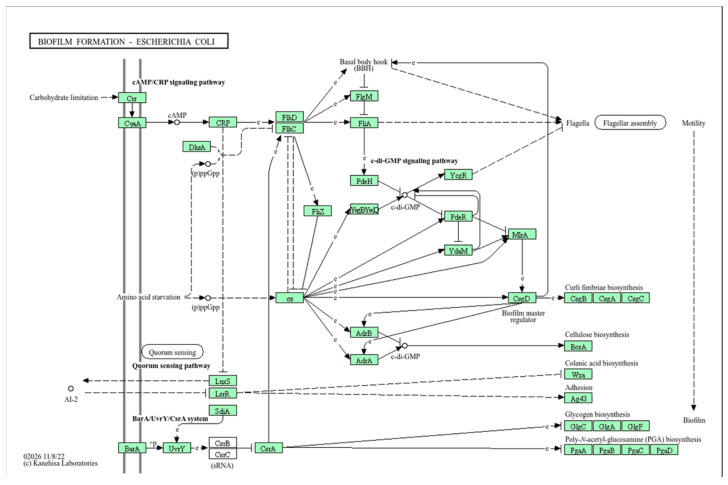
Pathways for biofilm formation. Functional genes in the genome of isolate GIMC1402:EC_33P15 are highlighted in green. The solid arrows indicate molecular interaction or relation, the dashed arrows indicate indirect link or unknown reaction; e—expression, +p—phosphorylation; circle—chemical compound.

Long-term persistence in the airways during inflammation has formed a hypermucoviscous phenotype of CF isolates (Figure 6). Colanic acid (CA) is the major exopolysaccharide produced by *E. coli*. CA forms slime, which increases cell viability and protects bacterial cells from environmental stress [61]. The up-regulation of the CA production is promoted via the global regulators LsrF and LsrK bound to AI-2. The production of the CA is a part of the process of colonization. In addition to colonization, AIs also promote aggregation, biofilm formation, and adherence. KEGG pathway maps confirm the presence of functional genes for the indicated processes in CF isolates (Figure 7).

As shown via BlastKOALA analysis, CF isolates can synthesize, export extracellularly, and internalize AI-2 and AI-3. The role of AI-2 in biofilm formation is shown in Figure 7. AI-3, in turn, via the QseBC system, regulates the expression of flagella and controls the formation of biofilm, influencing its thickness and architecture.

The next strategy for bacterial survival during chronic infection is to hijack and overcome the host’s antimicrobial responses and the effects of antibiotics used for treatment. This is realized by invading epithelial cells and macrophages for survival and replication, which is facilitated by two secretion systems: type 3 (T3SS) and type 6 (T6SS). The genomes of CF isolates contain T3SS, named *E. coli* type 3 secretion system 2 (ETT2) by Hayashi et al. [62]. The genes of the two regions encode components of the secretion apparatus. The EspL1 effector gene is located separately. The T6SS genes, with the exception of the genes encoding the tip protein VgrG, are concentrated in one chromosome cluster. The cluster includes, among other things, the *hcp1* and *hcp2* genes encoding the T6SS effectors.

We assessed the ability of isolates to invade and replicate in macrophages, as well as the efficiency of biofilm formation, via in vitro experiments.

### 2.3. In Vitro Experiments

#### 2.3.1. Biofilm Formation

**Figure 8:**
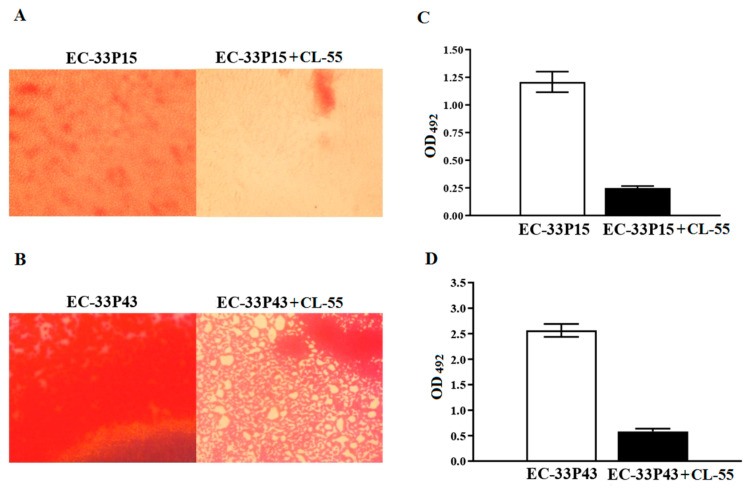
Biofilm formation via *E. coli* isolates in the absence and presence of the active pharmaceutical ingredient of Fluorthiazinone, CL-55, on polystyrene 96-well plates for 72 h. (**A**,**B**): photomicrograph of crystal violet-stained biofilm under light microscopy (200×). (**C**,**D**): the level of absorbance at 540 nm.

**Figure 9:**
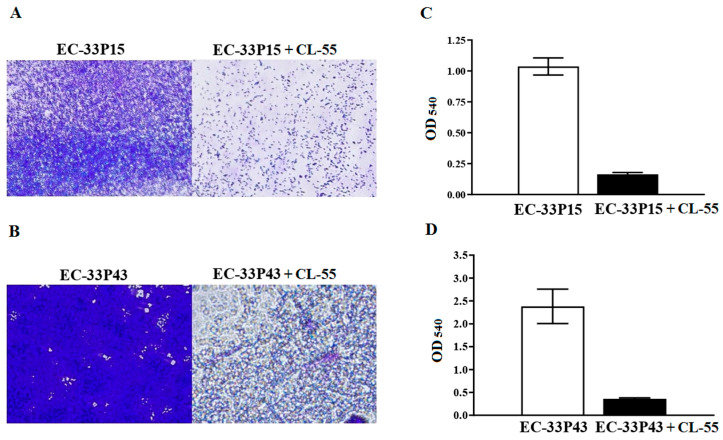
Biofilm formation via *E. coli* isolates in the absence and presence of the active pharmaceutical ingredient of Fluorthiazinone, CL-55, on polystyrene 96-well plates for 72 h. (**A**,**B**) photomicrograph of Congo red-stained biofilm under light microscopy (200×). (**C**,**D**) the level of absorbance at 492 nm.

Both isolates grew slowly due to the previously noted hypermucoviscous nature, which is consistent with the observation of Cui et al. that virulent pathogens grow more slowly because they can divert more energy towards other disease-specific processes, such as the production of virulence factors [63]. Therefore, the observation time for the ability of *E. coli* isolates to form biofilms on the abiotic surface of the 96-well plates was longer. After cultivation for 72 h, both isolates formed typical mature biofilms (Figure 8). However, the biofilm of the GIMC1403:EC_33P43 isolate was significantly denser according to the biomass (Figure 8) and matrix staining (Figure 9).

#### 2.3.2. Macrophage Internalization and Survival

**Figure 10:**
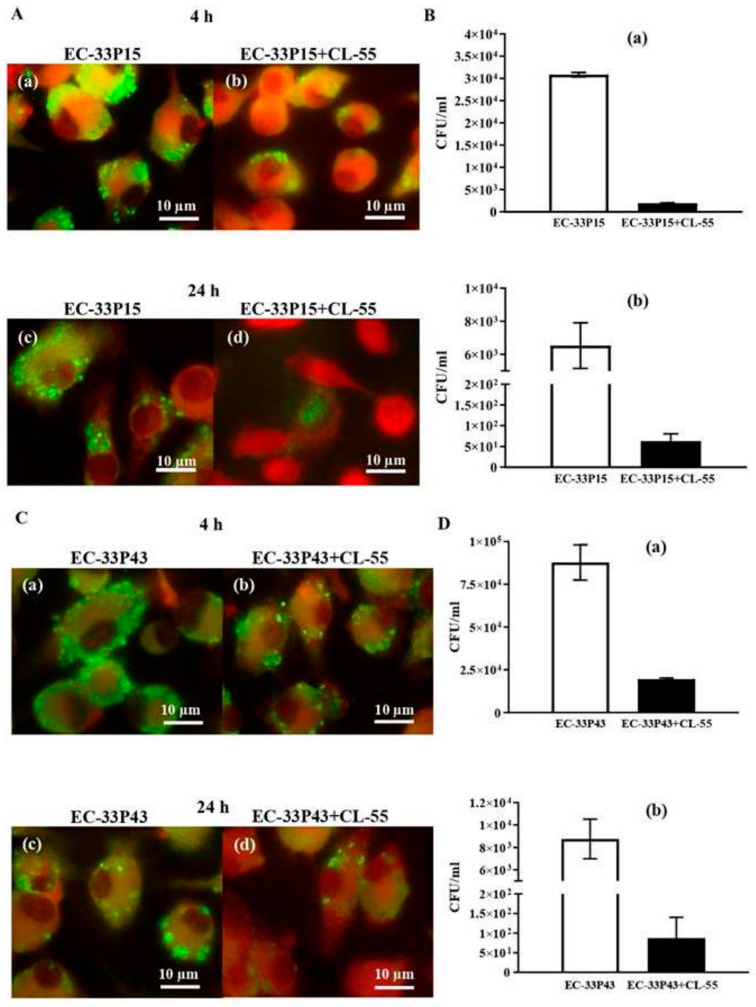
Bacterial survival in RAW264.7 macrophages 4 and 24 h after infection with *E. coli* cultures in the absence and presence of antibacterial drug CL-55 (the active pharmaceutical ingredient of Fluorthiazinone). (**A**,**C**) photomicrographs of the cell culture in the plate at 4 and 24 h of cultivation under a fluorescent microscope (1000×), respectively. (**A**(**a**,**c**)) and (**C**(**a**,**c**)) infected macrophages without CL-55 treatment; (**A**(**b**,**d**)) and (**C**(**b**,**d**)) infected macrophages treated with CL-55. RAW264.7 macrophages are stained with Evans blue (red). Bacteria labeled with indirect FITC fluorescent antibodies are green. Scale bar = 10 µm. (**B**,**D**) bacterial load in macrophages determined via serial plating of macrophage lysates. (**B**(**a**,**b**)) CFU of *E. coli* isolate GIMC1402:EC-33P15 after 4 and 24 h; (**D**(**a**,**b**)) CFU of *E. coli* isolate GIMC1403:EC-33P43 after 4 and 24 h.

Both GIMC1402:EC-33P15 and GIMC1403:EC_33P43 isolates were able to penetrate macrophages. Microscopy of anti-*E. coli* antibody-stained cell monolayer revealed bacterial intracellular communities inside macrophages at 4 and 24 h after interaction between bacteria and macrophages (Figure 10). When RAW264 macrophages were infected with GIMC1402:EC-33P15 at a multiplicity of infection (MOI) of 10 (5 × 10^6^ CFU/mL), the number of viable intracellular bacteria after 1 h was 3.4 × 10^4^ CFU/mL, and remained at the same number after 4 h (Figure 10A(a),B(a)). After 24 h, GIMC1402:EC-33P15 retained the ability to replicate in macrophages and was detected at a concentration of 6.5 × 10^3^ CFU/mL (Figure 10A(c),B(b)). The *E. coli* isolate GIMC1403:EC-33P43 used at MOI 10 formed an order of magnitude larger number of intracellular bacteria (3.5 × 10^5^ CFU/mL) at 1 and 4 h after capture via RAW264 macrophages (Figure 10C(a),D(a)). After 24 h of incubation, GIMC1402:EC-33P43 was detected in macrophages at an amount of 8.5 × 10^3^ CFU/mL (Figure 10C(c),D(b)).

Thus, both CF isolates demonstrated the ability to form biofilms and survive inside macrophages, which likely contributes to the long-term chronic respiratory tract infection of patients 119-CF and 149-CF, and the ineffectiveness of antibiotic and CFTR modulator therapy in reducing the bacterial load in the patients’ lungs. Therefore, we tested the sensitivity of CF isolates to the new antibacterial drug Fluorthiazinone (CL-55, the active pharmaceutical ingredient) in biofilms and during intracellular localization.

#### 2.3.3. CL-55 Does Not Inhibit Bacterial Viability

The results of a study of the direct antibacterial effect of CL-55 on *E. coli* CF isolates showed that the virulence inhibitor does not inhibit bacterial growth when cultured in vitro at concentrations from 25 to 300 μM.

#### 2.3.4. Effect of the Active Pharmaceutical Ingredient CL-55 on Biofilm Formation and Intracellular Survival of CF Isolates

Returning to Figure 8 and Figure 9, we can see that incubation of CF isolates with CL-55 leads to a decrease in both the amount of biomass and the density of the biofilm, and the suppression of the production of the exopolysaccharide matrix. The biofilm density showed a 6-fold decrease for the GIMC1402:EC-33P15 isolate and a 7-fold decrease for GIMC1403:EC_33P43 (Figure 8C,D). The production of the exopolysaccharide matrix showed a 5-fold decrease for the GIMC1402:EC-33P15 isolate and a 4-fold decrease for GIMC1403:EC_33P43 (Figure 9C,D).

The effect of CL-55 on CF isolates inside macrophages can be seen in Figure 10A(b,d),C(b,d)). The introduction of CL-55 simultaneously with *E. coli* GIMC1402:EC-33P15 infection of the RAW264.7 monolayer reduced the bacterial load in macrophages to 2.0 × 10^3^ CFU/mL and 1.9 × 10^3^ CFU/mL after 1 and 4 h, respectively (Figure 10B(a)). After 24 h, in the presence of CL-55, a significant decrease in viable intracellular bacteria to 6.3 × 10^1^ CFU/mL was observed (Figure 10B(b)).

For the *E. coli* isolate GIMC1403:EC-33P43, in the presence of CL-55, the number of intracellular bacteria was decreased by 1 order of magnitude after 1 and 4 h, and by 2 orders after 24 h (Figure 10C,D).

Thus, the new antibacterial drug exerted a suppressive effect on both the formation of biofilms via *E. coli* CF isolates and the proliferation of bacteria inside macrophages.

## 3. Discussion

Two isolates of *E. coli* ST648 were analyzed in this study. Both strains were from patients with long-term CF. The molecular confirmation of the *E. coli* genotype first occurred in 2013 and 2015. Patients’ time of infection via CTX-M-15-producing *E. coli* was roughly determined as no earlier than 2006 based on comparison of CF isolates with evolutionary clades of ST648 isolates. These clades were predicted by Schaufler et al. through Bayesian analysis of the population structure [7].

Whole-genome sequencing allowed comparison of chromosomes and episomes of two CF isolates, and confirmed the plasticity of the *E. coli* ST648 genomes. As a result of the recombination event, the K5 capsule type in GIMC1402:EC_33P15 is changed to the K4 type in GIMC1403:EC_33P43. Both capsules, heparosan-containing K5 and chrondodontin-containing K4, are associated with the ExPEC [64]. These capsule types mimic polysaccharides present in human tissue cells, making them poorly immunogenic but highly virulent [64,65]. Recombination events also affected the CRISPR region, indicating a different spectrum of bacteriophages with which the isolates could interact. Recombination also provided *E. coli* GIMC1402:EC_33P15 with the advantage of being able to utilize an additional carbon source, propanediol, in a specialized microcompartment.

MGE introduced AMP genes into both the chromosome and plasmid of isolate GIMC1402:EC_33P15, expanding the resistance potential compared to isolate GIMC1403:EC_33P43.

The ESBL genes, as markers of *E. coli* ST648, deserve special attention. Both isolates produced ESBL CTX-M-15 and OXA-1, but TEM-1 was a distinctive feature of isolate GIMC1402:EC_33P15. *E. coli* ST648’s history of acquiring ESBL genes also shows the genomic plasticity of isolates of this genotype. Let us consider clinical isolates. The first isolate ST648 was registered in the USA in 2007 and produced only ESBL CTX-M-15, as did the isolate in 2008 [14] and the isolate from Tanzania in 2011 [15]. The ESBL spectrum in the 2010 isolates from China [16] and 2015 isolates from Nepal [9] expanded to include CTX-M-15, OXA-1, and TEM-1, which corresponds to the ESBL set in the GIMC1402:EC_33P15 isolate. In 2011, an isolate from a patient returning to the UK after hospitalization in India produced NDM-5 along with CTX-M-15 and TEM-1 [17]. Finally, in 2023, isolates with CTX-M-15 and KPC-2 were recovered from patients in Argentina [10] and China [11]. The diversity of ESBLs is even greater in isolates from wild birds and farm animals. CTX-M of different alleles, CTX–M–2 [18], CTX–M–8 [19], or CTX–M–55 [20], can be supplemented with TEM-1 and NDM-5 (*Cairina moschata*) [18] or CMY–2 and AmpC (*Fregata magnificens*) [20]. Thus, MGE facilitates *E. coli* ST648 isolates’ accumulation of resistance genes.

In our study, *E. coli* GIMC1403:EC_33P43, which is less resistant in the planktonic state, has a protective mechanism that helps to avoid the action of antibiotics, an advantage for biofilm formation. The genome of GIMC1403:EC_33P43 encodes the regulator of biofilm formation, BssS, which is likely why the isolate GIMC1403:EC_33P43 forms a denser biofilm on the abiotic surface.

The second defense mechanism, survival and reproduction inside eukaryotic cells such as macrophages, worked equally well in two isolates.

Not all antibiotics are effective in killing bacteria inside eukaryotic cells. The lipophilic compounds are able to passively diffuse through the membrane, so their intracellular-to-plasma ratios are greater than 0.5 [66]. The hydrophilic compounds may enter the cells only when in the presence of specific carriers [67]; therefore, their intracellular-to-plasma ratios are less than 0.5 [66]. Antibiotics recommended for the treatment of *E. coli* infection belong to the beta-lactam subclasses: cephalosporins and carbapenems, and are hydrophilic compounds. The patients’ long-term history of antibacterial treatment confirms the ineffectiveness of these compounds in eradicating *E. coli*.

The new antibacterial agent used in our study, Fluorthiazinone with the active pharmaceutical ingredient CL-55 (C19H17F2N3O4S), is lipophilic. The presence of fluorine atoms enhances the lipophilicity of CL-55 [38]. The lipophilicity ensures rapid drug absorption and transport to tissues, such as the spleen, lungs, urinary bladder, and prostate, and penetration into eukaryotic cells [68]. The CL-55 doses of 50 µM and 100 µM were not toxic to Mccoy B cells (a hybrid cell line consisting of human synovial cells and mouse fibroblasts) and peritoneal macrophages, respectively, and were effective in suppressing the intracellular development of chlamydia and *Salmonella enterica* [38,39]. We used a dose of 47.5 µM to treat the RAW 264.7 macrophages infected with CF isolates of *E. coli*. The residual amount of *E. coli* was 6% of the control after 4 h and 1% after 24 h.

Intracellular bacteria’s resistance to lipophilic antibacterial compounds was studied by Garcia-Medina et al. Mouse tracheal epithelial cells infected with a mucoid strain of *Pseudomonas aeruginosa* isolated from a patient with cystic fibrosis were exposed to ciprofloxacin at a concentration of 1.2 mM, which killed planktonic cells [69]. After 24 h of incubation, the number of intracellular bacteria decreased 16.7-fold, i.e., incomplete antibiotic killing was observed [69]. CL-55 at a concentration of 47.5 μM (25 times lower) decreased intracellular *E. coli* 103-fold after 24 h of incubation.

Biofilms, as aggregated microbes surrounded by a self-produced matrix, either adhering to surfaces or located in tissues or secretions [43], are well known in microbiology. However, in medicine, Höiby, studying lung infection in cystic fibrosis, was the first to propose the concept of biofilm infection and its importance with respect to chronic infections [70]. Bacteria in biofilm show increased tolerance to antimicrobials and resist the host’s antimicrobial defenses [71]. Staudinger et al., using a model system involving gels as growth substrates for *P. aeruginosa*, revealed that aggregates were 100- to 1,000-fold less susceptible to killing by tobramycin at a concentration of 21.4 µM compared to dispersed cells [72]. To study the effect of CL-55 on *E. coli* aggregates, we used a biofilm model on an abiotic surface. CL-55 at a concentration of 238 µM suppressed biofilm formation and exopolysaccharide matrix production in both isolates, GIMC1402:EC_33P15 and GIMC1403:EC_33P43, despite the denser biofilm of the latter. Thus, CL-55 was effective against the two most resistant states of bacteria in lung infections: intracellular and aggregate.

## 4. Materials and Methods

### 4.1. Materials

Sputum samples were obtained from two CF patients, 119-CF and 149-CF, with severe lung disease who were in a clinical state without exacerbation and received maintenance therapy. Patient 149-CF had also been taking a dual CFTR modulator treatment, Lumacaftor/Ivacaftor, for 2.2 years. The age of patient 119-CF was 39 years, and that of patient 149-CF was 29 years. The duration of infection with *E. coli* ST648, confirmed via molecular genetic methods, was 10 years for patient 119-CF and 9 years for patient 149-CF at the time of isolation of the described strains of *E. coli* ST648.

The antibacterial drug Fluorthiazinone (FT) and its active pharmaceutical ingredient CL-55 (C19H17F2N3O4S) [42] were used for in vitro experiments. A 4.8 mM stock solution of CL-55 in 0.1 M sodium acetate, pH 7.0 ± 0.2, was used for the biofilm assay. When working with cell lines, a 26.1 mM stock CL-55 solution in dimethyl sulfoxide (DMSO) was used.

### 4.2. Methods

Bacteria isolation and cultivation

*E. coli* was isolated on 5% blood agar and Endo agar. The isolates GIMC1402:EC_33P15 and GIMC1403:EC_33P43 were cultured in Luria–Bertani (LB) broth at 37 °C.

Isolate identification

To identify the isolates, two targets were amplified: *16S rDNA* with primers [73] and *adk* with primers adkF-12 (in home elaborated) and adkR1 [74]; then, amplicons were sequenced as described in [75] and identified via Standard Nucleotide BLAST NCBI and the *Escherichia* typing database (URL: https://pubmlst.org/bigsdb?db=pubmlst_escherichia_seqdef, accessed on 5 May 2025).

Microbiome analysis

The sputum microbiome composition was determined via massively parallel sequencing of the *16S rDNA* gene amplicons with the Illumina platform. The results, deposited in GenBank (bioproject PRJNA717158), were analyzed using the Microbial Genomics Module of the CLC Genomic Workbench v.21.0.1 package (QIAGEN, Valencia, CA, USA). Greengenes v13_8 database with a similarity level of 97% was used for the determination of Operational Taxonomic Units (OTU). The Shannon entropy index and the coefficient of phylogenetic diversity (PD) were used to assess alpha diversity.

Whole-genome sequencing

The genomes of the strains GIMC1402:EC_33P15 and GIMC1403:EC_33P43 were sequenced on the Illumina platform. The NadPrep EZ DNA Library Preparation protocol (Nanodigmbio (Nanjing) Biotechnology Co., Ltd., Nanjing, China) and the KAPA HyperPlus Kit (F. Hoffmann-La Roche Ltd., Basel, Switzerland) protocol were used for the preparation of the library. Sequencing was performed on MiSeq and NextSeq 500/550 (Illumina, San Diego, CA, USA). Genomes were assembled using CLC Genomics Workbench v. 21 (QIAGEN) and SPAdes v. 3.13.0 (St. Petersburg genome assembler, St. Petersburg, Russia, URL: https://ablab.github.io/spades/, accessed on 5 May 2025) [76]. The coverage of genomes was 233.0x (GIMC1402:EC_33P15) and 224.0x (GIMC1403:EC_33P43). The software Rapid Annotations Subsystems Technology (RAST) and SEED [77] in version RASTtk (https://github.com/SEEDtk/RASTtk?ysclid=mf2yuy3t2m770309857/, accessed on 5 May 2025) and the NCBI Prokaryotic Genome Annotation Pipeline (PGAP) [78] were used for genome annotation. WGS data are available in GenBank: BioProject PRJNA561493, Accession Numbers: CP181181-CP181185 and CP181392-CP181397.

Genome analysis

For virulence factor identification, the VFDB (virulence factor database, http://www.mgc.ac.cn/VFs/, accessed on 5 May 2025) database [79], Pathogenwatch v21.0.0 (https://pathogen.watch/, accessed on 5 May 2025) and BlastKOALA (KEGG Orthology And Links Annotation, https://www.kegg.jp/blastkoala/, accessed on 5 May 2025) [80] were used. The spectrums of antimicrobial resistance genes were determined using the CARD (Comprehensive Antibiotic Resistance Database, https://card.mcmaster.ca/, accessed on 5 May 2025) resource [81] and BV-BRC (Bacterial and Viral Bioinformatics Resource Center, https://www.bv-brc.org/, accessed on 5 May 2025), formed based on PATRIC [82]. To clarify the annotation of beta-lactamases, the beta-lactamase database (BLDB, http://bldb.eu/, accessed on 5 May 2025) [83] was consulted.

The search for mobile elements and associated genes was performed using ISAbR-0.1.6 [84] and MGE MobileElementFinder v1.0.3 (https://cge.food.dtu.dk/services/MobileElementFinder/, accessed on 5 May 2025) [85]. PlasmidFinder 2.1 (https://cge.food.dtu.dk/services/PlasmidFinder/, accessed on 5 May 2025) [86] was applied to identify incompatibility groups (Inc.) of plasmid replicons. For rapid identification, annotation, and visualization of prophage sequences in bacterial genomes and plasmids, the PHASTEST web server (PHAge Search Tool with Enhanced Sequence Translation https://phastest.ca/, accessed on 5 May 2025) was used [87]. The CRISPRCasFinder program (https://crisprcas.i2bc.paris-saclay.fr/CrisprCasFinder/Index, accessed on 5 May 2025) [88] was applied for the detection of CRISPRs and *cas* genes.

To compare the nucleotide sequences and proteomes, the off-line BLAST-2.10.0+ program (https://ftp.ncbi.nlm.nih.gov/blast/executables/blast+/LATEST/, accessed on 5 May 2025) was used. Visualization of the results of comparative chromosome analysis was performed using the BRIG 0.95 program (BLAST Ring Image Generator, http://sourceforge.net/projects/brig/, accessed on 5 May 2025) [89]. Comparison of plasmid sequences was visualized using the Artemis Comparison Tool (Sanger, Cambridge, UK) [90].

The Create Average Nucleotide Identity Comparison tool of CLC Genomics Workbench (QIAGEN, Germantown, MD, USA) was used to calculate two measures: the Alignment Percentage (AP) and the Average Nucleotide Identity (ANI).

Phylogenetic analysis

Preliminary determination of the phylogenetic group affiliation of the analyzed isolates was performed based on the scheme proposed by Clermont O. et al. [91]. The analysis was performed in silico, and the corresponding gene fragments from the reference genomes, TspE4.C2 and *aceK* (CP161809.1), *chuA* (U67920.1), and *yjaA* (NC_000913.3), were compared using the BLAST-2.10.0+ program with the sequences of the complete genomes of the strains GIMC1402:EC_33P15 and GIMC1403:EC_33P43.

More complicated analysis included the alignment of concatenated nucleotide sequences of Achtman and Pasteur’s MLST scheme (except for the *uidA* gene) and the *aceK* gene fragment from the scheme proposed by Clermont O. et al. [91]; phylogenetic trees were constructed in the MEGA11 program [92]. *E. coli* genomes of different phylogenetic groups—A (NZ_CP033020, AP009048.1), B1 (NC_011748.1, CP101307.1), B2 (NZ_MIPU00000000, NZ_CP051263), C (NZ_NXOC00000000, NZ_NKDU00000000), D (NC_011751.1, ADBA00000000), E (NC_002695.2, AEXG00000000.1), and F (NC_011750.1, AEXF00000000.1)—were applied as references. For phylogenetic tree rooting, the type strain of *E. fergusonii* ATCC 35469 (NC_011740.1) was used as an outgroup.

Evaluation of the antibacterial effect of CL-55

A total of 50 μL of the overnight culture of *E. coli* isolates was added to test tubes with 5 mL of LB broth containing different concentrations of CL-55 (0, 25, 50, 100, 200, 300 μM) and cultured in a shaker incubator at 37 °C and 250 rpm for 20 h. The number of viable bacteria was determined via serial dilutions and seeding on LB agar. The results were taken into account after 24 h of cultivation at 37 °C.

Biofilm assay

In this study, 96-well polystyrene plates were used for static biofilm formation on an abiotic surface. Overnight culture of *E. coli* isolates was adjusted to OD600 = 0.5. Evaluation of the effectiveness of CL-55 on biofilm formation was carried out using concentrations of 238 µM. CL-55 was added simultaneously with *E. coli* GIMC1402:EC-33P15 and GIMC1403:EC-33P43 cultures. LB broth was used as a negative control, and the *E. coli* culture without CL-55 was used as a positive control. The plates were incubated at 37 °C for 72 h. Further, the wells were washed with phosphate-buffered saline (PBS) to remove the non-adherent cells. For biomass quantification, biofilm was stained with 0.1% crystal violet (CV) solution for 15 min. For extracellular matrix quantification, 0.1% Congo red (CR) was added to the wells and incubated for 30 min. In order to remove excess dye, the wells were rinsed three times with sterile distilled water. For quantitative analysis, the dye bound to the biofilm was dissolved in 96% ethanol. The wells were read using a Multiskan EX microplate absorbance reader for CV and CR dyes at wavelengths of 540 and 492 nm, respectively. For visual analysis, plates with fixed and CV- and CR-stained biofilms were examined under a Nikon Eclipse 50i light microscope (Nikon, Japan) at a magnification of 20×. Each *E. coli* isolate was tested in 3 replicates per experiment and in at least 3 experiments.

Macrophage cell culture and growth conditions

RAW 264.7, which is a macrophage cell line that was established from a tumor in a male mouse induced with the Abelson murine leukemia virus, was used. RAW264.7 cells were incubated in RPMI-1640 cell medium supplemented with 5% FBS without the addition of antibiotics at 37 °C with 5% CO_2_ for 24 h. After, cell counts were measured.

Bacterial intracellular survival in RAW264.7 macrophages

For infection of RAW264.7 macrophages, 24-well glass plates (d = 12 mm) were used. Macrophages were infected with *E. coli* isolates with an MOI of 10 relative to 1.3 × 10^5^ cells/mL macrophage cells. To evaluate the effect of CL-55 on the ability of the isolates of *E. coli* to penetrate and survive within macrophages, CL-55 was added to the wells at a final concentration of 47.5 µM simultaneously with infection. Incubation was carried out for 3 h at 37 °C and 5% CO_2_. Then, the plates were washed. Gentamicin at a concentration of 100 µg/mL was added to the wells and incubated for 1 h. The plates were then washed to remove planktonic cells and the gentamicin contained in the medium. Uninfected RAW264.7 cells were used as a negative control. Infected cells incubated without the addition of CL-55 served as a positive control. The results were then evaluated microscopically and via seeding on LB agar for 24 h. For microscopic analysis, slides were fixed with methanol, incubated with mouse antibodies to *E. coli* for 30 min at 37 °C and 5% CO_2_, and stained with anti-mouse IgG-FITC conjugate (Merck, Rahway, NJ, USA) for 30 min at 37 °C and 5% CO_2_. The study was performed under a fluorescence microscope Nikon Eclipse 50i (Nikon, Tokyo, Japan) at 1000× magnification under oil immersion. For quantitative characterization, infected macrophages with and without the addition of CL-55 were lysed with 0.25% Triton X-100 (Sigma-Aldrich, St. Louis, MO, USA). The lysates were then cultured on LB agar via serial dilutions. All quantitative analyses were performed in triplicate.

To evaluate the effect of CL-55 on the ability of the isolates of *E. coli* to survive and multiply within macrophages, CL-55 was added to the wells at a final concentration of 47.5 µM simultaneously with infection and 4 h later. Incubation was carried out for 24 h. One hour before the end of incubation, the plates were washed, and gentamicin was added to eliminate planktonic cells. The plates were then washed to remove planktonic cells and the gentamicin contained in the medium. Uninfected RAW264.7 cells were used as a negative control. Infected cells incubated without the addition of CL-55 served as a positive control. The results were then evaluated as described earlier.

## 5. Conclusions

The study of two hypermucoviscous *E. coli* isolates, representatives of the globally distributed clone ST648, demonstrated their high adaptability to long-term persistence. Invasiveness, biofilm formation, survival, and proliferation inside eukaryotic cells provide high resistance to the host immune system and recommended antibiotic therapies. Even the addition of CFTR modulators for more than 2 years did not ensure eradication of *E. coli* in patient 149-CF. The efficacy of Fluorthiazinone (CL-55) against *E. coli* ST648 CF isolates, both in aggregates and inside macrophages, demonstrated in in vitro experiments suggests the potential of further studies on the antibacterial activity of Fluorthiazinone against a range of *E. coli* genotypes and classical CF pathogens. Research on the invasion and intracellular survival of bacteria, not only in mouse models but also in human macrophages, will allow us to approximate clinical trials of FT in a cohort of patients with long-term CF.

## 6. Patents

Sputum samples from two adult patients (119-CF and 149-CF) were included in the study. The participants signed informed consent to take part in the study, and the research protocol was approved by the Biomedical Ethics Committee of the N.F. Gamaleya National Research Center for Epidemiology and Microbiology (protocol No. 59, 8 September 2023) and the Ethics Committee of the Research Institute of Pulmonology under FMBA of Russia (protocol No. 04-23, 24 April 2023, and protocol No. 07-24, 18 December 2024).

## Figures and Tables

**Table 1:** Main features of genome annotation of *E. coli* isolates.

Features	GIMC1402:EC_33P15	GIMC1403:EC_33P43
Chromosome, bp.	5,115,804	5,032,495
Genes (total)	5292	5154
CDSs (total)	5171	5032
Genes (RNA)	121	122
tRNAs	94	95
Pseudo Genes (total)	206	202
CRISPR Arrays	2	2
Plasmid	pEC_33P15-1 (134,688 bp)	pEC_33P43-1 (110,734 bp)
	pEC_33P15-2 (4237 bp)	pEC_33P43-2 (7176 bp)
	pEC_33P15-3 (4072 bp)	pEC_33P43-3 (2091 bp)
		pEC_33P43-4 (1459 bp)
Phage-Plasmid	p-pEC_33P15 (108,306 bp)	p-pEC_33P43 (107,320 bp)

CDS: coding DNA sequence.

**Table 2:** Resistance and virulence factors in pEC_33P15-1 and pEC_33P43-1.

Category	Function		GIMC1402:EC_33P15, pl1	GIMC1403:EC_33P43, pl1
			Product (Genome Position)	Product (Genome Position)
Resistance	Tetracycline resistance		Tetracycline efflux MFS transporter Tet(B) (50012..51217)	–
			Transcriptional repressor TetR(B) (51299..51922)	–
	Chloramphenicol resistance		CatA1, chloramphenicol O-acetyltransferase (54209..54868)	–
	Macrolide resistance		Mph(A), family macrolide 2′-phosphotransferase (59909..60814)	–
			Mrx(A), macrolide resistance MFS transporter (60811..62049)	–
	Sulfonamide resistance		Sul1 sulfonamide-resistant dihydropteroate synthase (66125..66964)	–
	Aminoglycoside resistance		AadA5, ANT(3′)-Ia family aminoglycoside nucleotidyltransferase (67511..68299)	–
	Trimethoprim resistance		Dft17, trimethoprim-resistant dihydrofolate reductase (68430..68903)	–
	Quaternary ammonium compound resistance		QacE, QAC efflux SMR transporter (66958..67305)	–
Virulence factors	Protection against the macroorganism’s complement system; participation in the biofilm formation		F-type transfer system (24233–34796; 79117–84085)	F-type transfer system (22132–41597)
	Colonization and survival under conditions of Fe^2+^, Pb^2+^, Zn^2+^, and Mn^2+^ deficiency		iucABCD, iutA, aerobactin (110608..119908)	–
			Fe^2+^ ABC-transporter (38945..43430)	Fe^2+^ ABC-transporter (59262..54777)
			Fe^2+^/Pb^2+^ ILT-transporter (43534..45988)	Fe^2+^/Pb^2+^ ILT-transporter (54673..52237)
			SitABCD ABC-transporter (105156..108605)	–
			–	TonB-dependent transport system (68082..71288; 81969..83939)
			–	YncE protein (71357..72532)
Toxin–antitoxin systems (TA)	Selective advantage of a clone in a bacterial population, formation of a persistent cell population	Type I *	Mok/Hok TA (20002..20218)	Mok/Hok TA (18537..18753)
			Hok/Gef TA (77769..77903)	Hok/Gef TA (42811..42945)
		Type II **	–	Phd_YefM/Fic_DOC TA (98859..99460)
			VapB/VapC TA (123886..124529; 128749..129392)	–
			CcdA/CcdB TA (133356..133881)	CcdA/CcdB TA (109402..109927)
			PemL/PemK TA (71821..72412)	PemL/PemK TA (46343..46934)

* The antitoxin is a small antisense RNA targeting toxin mRNA for degradation and/or inhibition of translation [46]. ** The antitoxin is a protein that forms a stable inactive complex with the toxin [46].

**Table 3:** Transposon regions containing AMR genes in the chromosomes of *E. coli* isolates.

Region	GIMC1402:EC_33P15		GIMC1403:EC_33P43	
	Gene	Product	Gene	Product
1	351708..350878	TEM-1		
	351852..352556	IS6-Tnp	331206..331280	IS6-Tnp-pseudo
	352700..353254	AAC(6′)-Ib-cr	331424..331978	AAC(6′)-Ib-cr
	353385..354215	OXA-1	332109..332939	OXA-1
	354353..354793	CatB3-pseudo	333077..333517	CatB3-pseudo
	complement (354847..355551)	IS6-Tnp	complement (333571..334275)	IS6-Tnp
	355658..356518	AAC(3)-IIa		
	356531..357073	tmrB		
	357165..358213	IS3-Tnp		
	complement (358267..358971)	IS6-Tnp		
2	complement (359039..361267)	Tn3-Tnp-pseudo	complement (334343..336571)	Tn3-Tnp-pseudo
	complement (361672..362547)	CTX-M-15	complement (336976..337851)	CTX-M-15
	complement (362803..364065)	IS1380-Tnp	complement (338107..339369)	IS1380-Tnp

**Table 4:** Main regions of differences in chromosomes of *E. coli* isolates.

Indel	GIMC1402:EC_33P15	GIMC1403:EC_33P43
**Indel 1**	the operon for ABC transporter complex UgpBAEC	no
**Indel 2**	4 ORFs, including the ORF of the small-membrane protein Blr	no
**Indel 3**	duplication of ORFs for the type IV toxin–antitoxin system	no
**Indel 4**	the 3rd operon for the tripartite ATP-independent periplasmic (TRAP) transporter	no
**Indel 5**	no	ORFs of the energy-coupling factor (ECF)–ABC transporter for cobalt transport
**Indel 6**	no	the genes for some metabolic pathways and an additional GntP family transporter (gluconate:H+ symporter)
**Indel 7**	no	11 ORFs, and the most important are the *mdtH* gene encoding the multidrug efflux MFS transporter, and the biofilm formation regulator BssS
**Region of original ORFs**	the K5 capsular gene cluster	the K4 capsular gene cluster
	ORFs for the metabolosome (bacterial microcompartment) organization and propanediol utilization	no

ORF: open reading frame.

**Table 5:** CRISPR in the chromosomes of *E. coli* isolates.

Position	CRISPR Length	Consensus_Repeat	Repeat ID (CRISPRdb)	Spacers Nb	Evidence Level
GIMC1402:EC_33P15					
1154150…1155279	1129	GTGTTCCCCGCGCCAGCGGGGATAAACCG	R6121	18	4
1180826…1181648	822	GAGTTCCCCGCGCCAGCGGGGATAAACCG	R3946	13	4
GIMC1403:EC_33P43					
1115575…1116213	638	GAGTTCCCCGCGCCAGCGGGGATAAACCG	R3946	10	4
1143351…1143925	574	GTGTTCCCCGCGCCAGCGGGGATAAA	Unknown	9	4

**Table 6:** The pairwise ANI (Average Nucleotide Identity) and AP (Alignment Percentage) values between study isolates and strains, representatives of clades 1–4 for *E. coli* ST648.

Strain, Accession number	GIMC1402:EC_33P15, CP181181.1	GIMC1403:EC_33P43, CP181392.1	NA023, JSXK000000000.1	32–2823 ED, DABAXP000000000.1	VB 962116, DABAMI000000000.1	F_30_1_R8, PIIR000000000.1
clade			1	2	3	4
GIMC1402:EC_33P15	-	99.1	**99.49**	**99.54**	98.92	99.08
GIMC1403:EC_33P43	95.65	-	**99.39**	**99.36**	98.84	98.98
NA023, clade 1	**92.45**	**91.54**	-	99.51	98.98	98.98
32–2823 ED, clade 2	89.32	89.96	90.07	-	99.33	99.43
VB 962116, clade 3	88.07	87.82	98.98	90.01	-	99.72
F_30_1_R8, clade 4	90.63	89.98	89.95	89.76	90.87	-

The upper right triangle shows the ANI values (%); the lower left triangle shows the AP values (%). The *E. coli* ST648 clades were determined according to Schaufler et al. [7].

## Data Availability

The reported results can be found in the GenBank. Accession Numbers: CP181181-CP181185, CP181392-CP181397, and bioproject PRJNA717158.

## Abbreviations

The following abbreviations are used in this manuscript:

ANI	Average nucleotide identity
AP	Alignment percentage
CA	Colanic acid
CAP	Community-acquired pneumonia
CDS	Coding DNA sequence
CF	Cystic fibrosis
CL-55	The active pharmaceutical ingredient of Fluorthiazinone
CPS	Capsular polysaccharide
CRISPR	Clustered regularly interspaced short palindromic repeats
ECF	Energy-coupling factor
ESBL	Extended-spectrum beta-lactamase
ETT2	E. coli type 3 secretion system 2
ExPEC	Extraintestinal pathogenic *E. coli*
FT	Fluorthiazinone
MOI	Multiplicity of infection (the ratio of the number of bacterial cells to the number of host cells)
NP	Nosocomial pneumonia
ORF	Open reading frame
P-P	Phage-plasmid
T3SS	Type 3 secretion system
T6SS	Type 6 secretion system
TRAP	Tripartite ATP-independent periplasmic transporter
VAP	Ventilator-associated pneumonia
